# Aqua­(1,10-phenanthroline-κ^2^ *N*,*N*′)(dl-threoninato-κ^2^ *N*,*O* ^1^)copper(II) chloride dihydrate

**DOI:** 10.1107/S1600536810015278

**Published:** 2010-04-30

**Authors:** Yi-Han Tan, Siang-Guan Teoh, Kei-Lin Sek, Wan-Sin Loh, Hoong-Kun Fun

**Affiliations:** a School of Chemical Sciences, Universiti Sains Malaysia, 11800 USM, Penang, Malaysia; b X-ray Crystallography Unit, School of Physics, Universiti Sains Malaysia, 11800 USM, Penang, Malaysia

## Abstract

The asymmetric unit of the title compound, [Cu(C_4_H_8_NO_3_)(C_12_H_8_N_2_)(H_2_O)]Cl·2H_2_O, contains a complex cation, a chloride anion and two water mol­ecules. The Cu^II^ ion has a distorted square-pyramidal coordination geometry formed by one bidentate phenanthroline ligand, one *O*,*N*-bidentate dl-threoninate ligand and one apical water mol­ecule. In the crystal structure, inter­molecular O—H⋯O, N—H⋯O, N—H⋯Cl and O—H⋯Cl hydrogen bonds link the components into layers. A single weak inter­molecular C—H⋯O inter­action connects these layers into a three-dimensional network.

## Related literature

For background to the inter­actions of transition-metal complexes with DNA, see: Vaidyanathan & Nair (2003 ▶); Rao *et al.* (2007 ▶, 2008 ▶); Kumar & Arunachalam (2007 ▶); Patel *et al.* (2006 ▶); Wang *et al.* (2007 ▶); Zhang *et al.* (2004 ▶). For a related structure, see: Lu *et al.* (2004 ▶). For standard bond-length data, see: Allen *et al.* (1987 ▶).
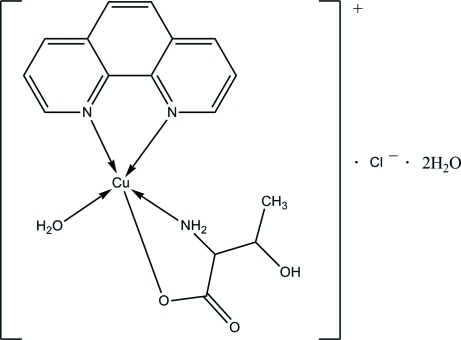


## Experimental

### 

#### Crystal data

[Cu(C_4_H_8_NO_3_)(C_12_H_8_N_2_)(H_2_O)]Cl·2H_2_O
*M* *_r_* = 451.36
Triclinic, 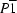

*a* = 7.1972 (1) Å
*b* = 11.9785 (2) Å
*c* = 12.2915 (2) Å
α = 65.664 (1)°
β = 78.079 (1)°
γ = 81.345 (1)°
*V* = 942.15 (3) Å^3^
*Z* = 2
Mo *K*α radiation
μ = 1.34 mm^−1^
*T* = 296 K
0.34 × 0.20 × 0.07 mm

#### Data collection

Bruker APEXII DUO CCD area-detector diffractometer
Absorption correction: multi-scan (*SADABS*; Bruker, 2009 ▶) *T*_min_ = 0.656, *T*_max_ = 0.911
29845 measured reflections
8056 independent reflections
5995 reflections with *I* > 2σ(*I*)
*R*_int_ = 0.028

#### Refinement

*R*[*F* ^2^ > 2σ(*F* ^2^)] = 0.036
*wR*(*F* ^2^) = 0.092
*S* = 1.04
8056 reflections
245 parameters
H-atom parameters constrained
Δρ_max_ = 0.47 e Å^−3^
Δρ_min_ = −0.41 e Å^−3^

### 

Data collection: *APEX2* (Bruker, 2009 ▶); cell refinement: *SAINT* (Bruker, 2009 ▶); data reduction: *SAINT*; program(s) used to solve structure: *SHELXTL* (Sheldrick, 2008 ▶); program(s) used to refine structure: *SHELXTL*; molecular graphics: *SHELXTL*; software used to prepare material for publication: *SHELXTL* and *PLATON* (Spek, 2009 ▶).

## Supplementary Material

Crystal structure: contains datablocks global, I. DOI: 10.1107/S1600536810015278/lh5025sup1.cif

Structure factors: contains datablocks I. DOI: 10.1107/S1600536810015278/lh5025Isup2.hkl

Additional supplementary materials:  crystallographic information; 3D view; checkCIF report

## Figures and Tables

**Table 1:** Hydrogen-bond geometry (Å, °)

*D*—H⋯*A*	*D*—H	H⋯*A*	*D*⋯*A*	*D*—H⋯*A*
O1*W*—H1*W*1⋯Cl1	0.80	2.36	3.1411 (13)	166
O1*W*—H2*W*1⋯O2^i^	0.82	1.90	2.7114 (18)	167
O3—H1*O*3⋯O1^ii^	0.90	2.03	2.9089 (18)	164
N3—H1*N*3⋯Cl1^iii^	0.86	2.62	3.3992 (13)	151
N3—H2*N*3⋯O2*W*^i^	0.94	2.07	3.0085 (19)	175
O2*W*—H1*W*2⋯Cl1^i^	0.87	2.35	3.2104 (16)	175
O2*W*—H2*W*2⋯Cl1^iv^	0.84	2.33	3.1463 (14)	162
O3*W*—H1*W*3⋯O2*W*	1.01	1.95	2.954 (2)	170
O3*W*—H2*W*3⋯O3^ii^	0.91	2.03	2.901 (2)	159
C7—H7*A*⋯O2^v^	0.93	2.41	3.292 (2)	157

Symmetry codes: (i) 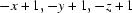; (ii) 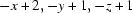; (iii) 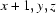; (iv) 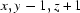; (v) 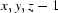.

**Fig. 1.**
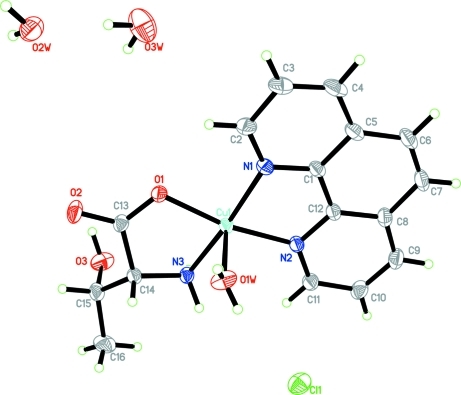
The molecular structure of the title compound, showing 50% probability displacement ellipsoids and the atom-numbering scheme.

**Fig. 2.**
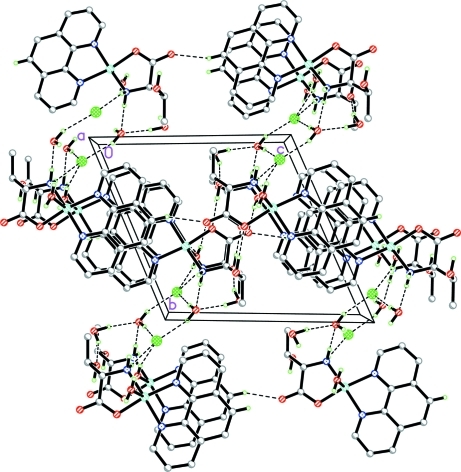
The crystal packing of the title compound, viewed along the a axis. Intermolecular interactions are shown as dashed lines. H atoms not involved in the intermolecular interactions (dashed lines) have been omitted for clarity.

## supplementary crystallographic information

### Comment

The interaction of transition metal complexes with DNA is a vibrant area of research and has long been investigated in relation to the development of new reagents for molecular biology, biotechnology and medicine (Vaidyanathan *et al.*, 2003; Rao *et al.*, 2008; Kumar *et al.*, 2007). Among all the transition metals, copper is the most widely used metals in these studies as it is a bioessential element with +1 and +2 oxidation states (Patel *et al.*, 2006; Wang *et al.*, 2007; Vaidyanathan *et al.*, 2003). Copper(II) complexes have been found to be useful in the treatment of many diseases as well as cancer. Copper(II) complexes of 1,10-phenanthroline and its derivatives exhibit various biological activities such as antimicrobial, antimycobaterial, anticandida and antitumor activities. Copper complexes of amino acids have been reported to exhibit effective antitumor and artificial nuclease activity. Several reports have also shown that these complexes show efficient DNA cleavage activity by either oxidative or hydrolytic pathways (Kumar *et al.*, 2007; Zhang *et al.*, 2004; Rao *et al.*, 2007). In the title compound, aqua(DL-threoninato-κ^2^*N,O*)(1,10-phenanthroline)copper(II) chloride dihydrate, DL-threonine has been selected as the ligand for the complex.

The asymmetric unit of the title compound (Fig. 1) consists of one Cu^II^ complex cation, one chlorine anion and two water molecules. The Cu^II^ ion is coordinated by N1 and N2 atoms from the phenanthroline ligand and N3 and O1 atoms from the threoninato ligand in the basal plane and the O1W water molecule is coordinated in the apical site to form a distorted square-pyramidal geometry. The bond lengths are within normal values (Allen *et al.*, 1987) and are comparable to those observed for a closely related structure (Lu *et al.*, 2004).

In the crystal structure (Fig. 2), intermolecular C7—H7A···O2 hydrogen bonds (Table 1) link the Cu^II^ complex cations into chains along the *c* axis. Intermolecular O1W—H2W1···O2, O3—H1O3···O1, N3—H2N3···O2W, O3W—H2W3···O3, O3W—H1W3···O2W, N3—H1N3···Cl1, O2W—H1W2···Cl1, O2W—H2W2···Cl1 and O1W—H1W1—Cl1 interactions (Table 1) link the molecules into a three-dimensional network.

### Experimental

To an ethanolic solution (10.0 ml) of copper(II) chloride dihydrate (0.1708 g, 1 mmol), an ethanolic solution (10.0 ml) of DL-threonine (0.1191 g, 1 mmol) was added. After a few minutes, an ethanolic solution (20.0 ml) of 1,10-phenanthroline (0.1982 g, 1 mmol) was added dropwise to the mixture solution. The pH of the resulting solution was then adjusted to pH 8 by adding a few drops of NaOH aqueous solution. The blue solution was filtered and left to evaporate slowly at room temperature. Blue blocky single crystals of the title compound suitable for X-ray diffraction were obtained after a few days.

### Refinement

H atoms attached to N and O atoms were located from difference Fourier map and allowed to ride on their parent atoms and constrained to be 1.5*U*_eq_ for the water molecules and 1.2*U*_eq_ for the amino group. The remaining H atoms were positioned geometrically and refined using a riding model, with *U*_iso_(H) = 1.2 or 1.5 *U*_eq_(C). A rotating-group model was applied for the methyl group [C–H = 0.93 to 0.98 Å, O–H = 0.7992 to 1.0137 Å, N–H = 0.8636 to 0.9420 Å].

### Crystal data

**Table:**

[Cu(C_4_H_8_NO_3_)(C_12_H_8_N_2_)(H_2_O)]Cl·2H_2_O	*Z* = 2
*M**_r_* = 451.36	*F*(000) = 466
Triclinic, *P*1	*D*_x_ = 1.591 Mg m^−^^3^
Hall symbol: -P 1	Mo *K*α radiation, λ = 0.71073 Å
*a* = 7.1972 (1) Å	Cell parameters from 9968 reflections
*b* = 11.9785 (2) Å	θ = 2.9–32.7°
*c* = 12.2915 (2) Å	µ = 1.34 mm^−^^1^
α = 65.664 (1)°	*T* = 296 K
β = 78.079 (1)°	Block, blue
γ = 81.345 (1)°	0.34 × 0.20 × 0.07 mm
*V* = 942.15 (3) Å^3^	

### Data collection

**Table:**

Bruker APEXII DUO CCD area-detector diffractometer	8056 independent reflections
Radiation source: fine-focus sealed tube	5995 reflections with *I* > 2σ(*I*)
graphite	*R*_int_ = 0.028
φ and ω scans	θ_max_ = 34.7°, θ_min_ = 1.8°
Absorption correction: multi-scan (*SADABS*; Bruker, 2009)	*h* = −11→11
*T*_min_ = 0.656, *T*_max_ = 0.911	*k* = −18→19
29845 measured reflections	*l* = −19→19

### Refinement

**Table:**

Refinement on *F*^2^	Primary atom site location: structure-invariant direct methods
Least-squares matrix: full	Secondary atom site location: difference Fourier map
*R*[*F*^2^ > 2σ(*F*^2^)] = 0.036	Hydrogen site location: inferred from neighbouring sites
*wR*(*F*^2^) = 0.092	H-atom parameters constrained
*S* = 1.04	*w* = 1/[σ^2^(*F*_o_^2^) + (0.0404*P*)^2^ + 0.1542*P*] where *P* = (*F*_o_^2^ + 2*F*_c_^2^)/3
8056 reflections	(Δ/σ)_max_ < 0.001
245 parameters	Δρ_max_ = 0.47 e Å^−^^3^
0 restraints	Δρ_min_ = −0.41 e Å^−^^3^

### Special details

**Table:**

Geometry. All esds (except the esd in the dihedral angle between two l.s. planes) are estimated using the full covariance matrix. The cell esds are taken into account individually in the estimation of esds in distances, angles and torsion angles; correlations between esds in cell parameters are only used when they are defined by crystal symmetry. An approximate (isotropic) treatment of cell esds is used for estimating esds involving l.s. planes.
Refinement. Refinement of *F*^2^ against ALL reflections. The weighted *R*-factor wR and goodness of fit *S* are based on *F*^2^, conventional *R*-factors *R* are based on *F*, with *F* set to zero for negative *F*^2^. The threshold expression of *F*^2^ > σ(*F*^2^) is used only for calculating *R*-factors(gt) etc. and is not relevant to the choice of reflections for refinement. *R*-factors based on *F*^2^ are statistically about twice as large as those based on *F*, and *R*- factors based on ALL data will be even larger.

### Fractional atomic coordinates and isotropic or equivalent isotropic displacement parameters (Å^2^)

**Table:**

	*x*	*y*	*z*	*U*_iso_*/*U*_eq_	
Cu1	0.76442 (2)	0.603112 (14)	0.250328 (14)	0.02828 (5)	
O1W	0.45829 (16)	0.66307 (10)	0.28057 (11)	0.0473 (3)	
H1W1	0.3972	0.7227	0.2422	0.071*	
H2W1	0.3770	0.6135	0.3201	0.071*	
O1	0.77687 (16)	0.50694 (9)	0.42081 (9)	0.0359 (2)	
O2	0.7643 (2)	0.52774 (11)	0.59298 (9)	0.0493 (3)	
O3	1.07351 (19)	0.74151 (11)	0.44492 (13)	0.0558 (3)	
H1O3	1.0957	0.6609	0.4891	0.084*	
N1	0.72609 (17)	0.45456 (11)	0.22337 (11)	0.0325 (2)	
N2	0.80300 (17)	0.67993 (11)	0.06759 (10)	0.0305 (2)	
N3	0.83142 (17)	0.74053 (10)	0.28305 (10)	0.0296 (2)	
H1N3	0.9497	0.7564	0.2596	0.036*	
H2N3	0.7570	0.8122	0.2433	0.036*	
C1	0.74069 (19)	0.47692 (13)	0.10467 (13)	0.0314 (3)	
C2	0.6821 (2)	0.34333 (14)	0.30518 (16)	0.0407 (3)	
H2A	0.6704	0.3275	0.3869	0.049*	
C3	0.6532 (3)	0.24997 (15)	0.27161 (19)	0.0491 (4)	
H3A	0.6223	0.1733	0.3307	0.059*	
C4	0.6702 (3)	0.27118 (16)	0.15218 (19)	0.0484 (4)	
H4A	0.6526	0.2088	0.1296	0.058*	
C5	0.7146 (2)	0.38811 (15)	0.06321 (16)	0.0394 (3)	
C6	0.7318 (2)	0.42233 (18)	−0.06444 (17)	0.0482 (4)	
H6A	0.7168	0.3641	−0.0932	0.058*	
C7	0.7694 (2)	0.53790 (18)	−0.14407 (16)	0.0467 (4)	
H7A	0.7796	0.5575	−0.2265	0.056*	
C8	0.7939 (2)	0.63054 (16)	−0.10411 (14)	0.0381 (3)	
C9	0.8273 (2)	0.75297 (17)	−0.18067 (14)	0.0454 (4)	
H9A	0.8351	0.7788	−0.2638	0.054*	
C10	0.8481 (2)	0.83416 (16)	−0.13287 (14)	0.0437 (4)	
H10A	0.8698	0.9154	−0.1833	0.052*	
C11	0.8368 (2)	0.79473 (14)	−0.00808 (13)	0.0364 (3)	
H11A	0.8534	0.8506	0.0232	0.044*	
C12	0.78101 (19)	0.59920 (14)	0.01996 (13)	0.0309 (3)	
C13	0.7721 (2)	0.57113 (13)	0.48255 (12)	0.0322 (3)	
C14	0.7668 (2)	0.71093 (13)	0.41344 (12)	0.0319 (3)	
H14A	0.6330	0.7421	0.4225	0.038*	
C15	0.8735 (3)	0.77207 (14)	0.46657 (14)	0.0409 (3)	
H15A	0.8300	0.7424	0.5541	0.049*	
C16	0.8433 (3)	0.91054 (16)	0.4121 (2)	0.0563 (5)	
H16A	0.9172	0.9447	0.4461	0.084*	
H16B	0.7109	0.9346	0.4297	0.084*	
H16C	0.8827	0.9403	0.3261	0.084*	
Cl1	0.22654 (6)	0.87371 (4)	0.09619 (4)	0.04803 (10)	
O2W	0.4269 (2)	0.03917 (12)	0.83732 (12)	0.0539 (3)	
H1W2	0.5174	0.0603	0.8600	0.081*	
H2W2	0.3827	−0.0182	0.9014	0.081*	
O3W	0.6557 (2)	0.08312 (18)	0.59634 (17)	0.0851 (5)	
H1W3	0.5665	0.0630	0.6763	0.128*	
H2W3	0.7213	0.1408	0.6002	0.128*	

### Atomic displacement parameters (Å^2^)

**Table:**

	*U*^11^	*U*^22^	*U*^33^	*U*^12^	*U*^13^	*U*^23^
Cu1	0.03672 (10)	0.02541 (8)	0.02496 (8)	−0.00530 (6)	−0.00529 (6)	−0.01097 (6)
O1W	0.0328 (5)	0.0366 (6)	0.0579 (7)	−0.0045 (4)	−0.0043 (5)	−0.0048 (5)
O1	0.0538 (6)	0.0256 (4)	0.0297 (5)	−0.0058 (4)	−0.0116 (4)	−0.0091 (4)
O2	0.0797 (9)	0.0429 (6)	0.0247 (5)	−0.0253 (6)	−0.0091 (5)	−0.0056 (4)
O3	0.0573 (8)	0.0389 (6)	0.0764 (9)	−0.0043 (5)	−0.0328 (7)	−0.0172 (6)
N1	0.0361 (6)	0.0288 (6)	0.0358 (6)	−0.0036 (5)	−0.0063 (5)	−0.0153 (5)
N2	0.0347 (6)	0.0314 (6)	0.0278 (5)	−0.0040 (5)	−0.0045 (4)	−0.0138 (4)
N3	0.0370 (6)	0.0273 (5)	0.0246 (5)	−0.0077 (4)	−0.0041 (4)	−0.0088 (4)
C1	0.0290 (6)	0.0341 (7)	0.0391 (7)	0.0011 (5)	−0.0089 (5)	−0.0221 (6)
C2	0.0470 (8)	0.0300 (7)	0.0449 (8)	−0.0055 (6)	−0.0078 (7)	−0.0137 (6)
C3	0.0521 (10)	0.0301 (7)	0.0664 (12)	−0.0059 (7)	−0.0099 (9)	−0.0192 (8)
C4	0.0465 (9)	0.0380 (8)	0.0762 (13)	0.0006 (7)	−0.0169 (9)	−0.0357 (9)
C5	0.0348 (7)	0.0408 (8)	0.0566 (10)	0.0045 (6)	−0.0136 (7)	−0.0326 (7)
C6	0.0465 (9)	0.0598 (11)	0.0623 (11)	0.0067 (8)	−0.0192 (8)	−0.0464 (10)
C7	0.0465 (9)	0.0663 (11)	0.0438 (9)	0.0066 (8)	−0.0156 (7)	−0.0378 (9)
C8	0.0339 (7)	0.0530 (9)	0.0345 (7)	0.0040 (6)	−0.0100 (6)	−0.0246 (7)
C9	0.0470 (9)	0.0588 (10)	0.0288 (7)	0.0008 (8)	−0.0080 (6)	−0.0165 (7)
C10	0.0497 (9)	0.0430 (8)	0.0308 (7)	−0.0044 (7)	−0.0045 (6)	−0.0076 (6)
C11	0.0421 (8)	0.0338 (7)	0.0319 (7)	−0.0060 (6)	−0.0033 (6)	−0.0118 (6)
C12	0.0274 (6)	0.0383 (7)	0.0324 (6)	0.0009 (5)	−0.0066 (5)	−0.0194 (6)
C13	0.0390 (7)	0.0303 (6)	0.0266 (6)	−0.0096 (5)	−0.0058 (5)	−0.0080 (5)
C14	0.0401 (7)	0.0293 (6)	0.0275 (6)	−0.0050 (5)	−0.0042 (5)	−0.0120 (5)
C15	0.0605 (10)	0.0353 (7)	0.0331 (7)	−0.0103 (7)	−0.0100 (7)	−0.0161 (6)
C16	0.0703 (12)	0.0361 (8)	0.0740 (13)	−0.0048 (8)	−0.0184 (10)	−0.0296 (9)
Cl1	0.0520 (2)	0.03659 (19)	0.0500 (2)	−0.01029 (17)	−0.01205 (18)	−0.00742 (17)
O2W	0.0616 (8)	0.0483 (7)	0.0475 (7)	−0.0081 (6)	−0.0133 (6)	−0.0114 (6)
O3W	0.0749 (11)	0.1128 (15)	0.0905 (13)	−0.0229 (10)	−0.0039 (9)	−0.0616 (12)

### Geometric parameters (Å, °)

**Table:**

Cu1—O1	1.9450 (10)	C4—H4A	0.9300
Cu1—N3	1.9921 (11)	C5—C6	1.432 (2)
Cu1—N1	2.0059 (12)	C6—C7	1.354 (3)
Cu1—N2	2.0210 (11)	C6—H6A	0.9300
Cu1—O1W	2.2167 (11)	C7—C8	1.431 (2)
O1W—H1W1	0.7992	C7—H7A	0.9300
O1W—H2W1	0.8250	C8—C12	1.398 (2)
O1—C13	1.2770 (17)	C8—C9	1.403 (3)
O2—C13	1.2295 (17)	C9—C10	1.366 (2)
O3—C15	1.429 (2)	C9—H9A	0.9300
O3—H1O3	0.9002	C10—C11	1.395 (2)
N1—C2	1.3333 (19)	C10—H10A	0.9300
N1—C1	1.3545 (19)	C11—H11A	0.9300
N2—C11	1.3298 (18)	C13—C14	1.5326 (19)
N2—C12	1.3615 (17)	C14—C15	1.524 (2)
N3—C14	1.4778 (17)	C14—H14A	0.9800
N3—H1N3	0.8636	C15—C16	1.511 (2)
N3—H2N3	0.9420	C15—H15A	0.9800
C1—C5	1.4047 (19)	C16—H16A	0.9600
C1—C12	1.435 (2)	C16—H16B	0.9600
C2—C3	1.397 (2)	C16—H16C	0.9600
C2—H2A	0.9300	O2W—H1W2	0.8654
C3—C4	1.365 (3)	O2W—H2W2	0.8422
C3—H3A	0.9300	O3W—H1W3	1.0137
C4—C5	1.410 (3)	O3W—H2W3	0.9153
			
O1—Cu1—N3	84.44 (4)	C7—C6—H6A	119.4
O1—Cu1—N1	92.20 (5)	C5—C6—H6A	119.4
N3—Cu1—N1	173.26 (5)	C6—C7—C8	121.40 (15)
O1—Cu1—N2	167.52 (5)	C6—C7—H7A	119.3
N3—Cu1—N2	99.88 (5)	C8—C7—H7A	119.3
N1—Cu1—N2	82.23 (5)	C12—C8—C9	116.68 (14)
O1—Cu1—O1W	94.75 (5)	C12—C8—C7	118.61 (16)
N3—Cu1—O1W	89.99 (5)	C9—C8—C7	124.70 (15)
N1—Cu1—O1W	96.12 (5)	C10—C9—C8	119.91 (14)
N2—Cu1—O1W	96.94 (5)	C10—C9—H9A	120.0
Cu1—O1W—H1W1	131.4	C8—C9—H9A	120.0
Cu1—O1W—H2W1	121.8	C9—C10—C11	119.74 (15)
H1W1—O1W—H2W1	103.0	C9—C10—H10A	120.1
C13—O1—Cu1	114.26 (9)	C11—C10—H10A	120.1
C15—O3—H1O3	108.0	N2—C11—C10	122.16 (14)
C2—N1—C1	118.50 (13)	N2—C11—H11A	118.9
C2—N1—Cu1	128.75 (11)	C10—C11—H11A	118.9
C1—N1—Cu1	112.69 (9)	N2—C12—C8	123.41 (14)
C11—N2—C12	118.07 (12)	N2—C12—C1	116.42 (12)
C11—N2—Cu1	129.83 (10)	C8—C12—C1	120.17 (13)
C12—N2—Cu1	112.02 (9)	O2—C13—O1	123.99 (13)
C14—N3—Cu1	106.69 (8)	O2—C13—C14	119.04 (13)
C14—N3—H1N3	113.4	O1—C13—C14	116.91 (11)
Cu1—N3—H1N3	114.4	N3—C14—C15	114.04 (12)
C14—N3—H2N3	105.0	N3—C14—C13	109.43 (11)
Cu1—N3—H2N3	108.9	C15—C14—C13	112.30 (12)
H1N3—N3—H2N3	108.0	N3—C14—H14A	106.9
N1—C1—C5	123.26 (14)	C15—C14—H14A	106.9
N1—C1—C12	116.62 (12)	C13—C14—H14A	106.9
C5—C1—C12	120.10 (14)	O3—C15—C16	107.21 (14)
N1—C2—C3	121.86 (16)	O3—C15—C14	110.23 (13)
N1—C2—H2A	119.1	C16—C15—C14	112.68 (14)
C3—C2—H2A	119.1	O3—C15—H15A	108.9
C4—C3—C2	120.03 (16)	C16—C15—H15A	108.9
C4—C3—H3A	120.0	C14—C15—H15A	108.9
C2—C3—H3A	120.0	C15—C16—H16A	109.5
C3—C4—C5	119.66 (15)	C15—C16—H16B	109.5
C3—C4—H4A	120.2	H16A—C16—H16B	109.5
C5—C4—H4A	120.2	C15—C16—H16C	109.5
C1—C5—C4	116.68 (15)	H16A—C16—H16C	109.5
C1—C5—C6	118.46 (15)	H16B—C16—H16C	109.5
C4—C5—C6	124.85 (15)	H1W2—O2W—H2W2	101.9
C7—C6—C5	121.24 (15)	H1W3—O3W—H2W3	98.8
			
N3—Cu1—O1—C13	16.30 (10)	C1—C5—C6—C7	−1.0 (2)
N1—Cu1—O1—C13	−169.56 (10)	C4—C5—C6—C7	178.03 (17)
N2—Cu1—O1—C13	127.31 (19)	C5—C6—C7—C8	0.1 (3)
O1W—Cu1—O1—C13	−73.23 (10)	C6—C7—C8—C12	0.9 (2)
O1—Cu1—N1—C2	13.31 (14)	C6—C7—C8—C9	−177.91 (16)
N2—Cu1—N1—C2	−177.92 (14)	C12—C8—C9—C10	1.0 (2)
O1W—Cu1—N1—C2	−81.72 (14)	C7—C8—C9—C10	179.83 (16)
O1—Cu1—N1—C1	−169.68 (10)	C8—C9—C10—C11	0.2 (3)
N2—Cu1—N1—C1	−0.91 (10)	C12—N2—C11—C10	0.7 (2)
O1W—Cu1—N1—C1	95.29 (10)	Cu1—N2—C11—C10	−175.89 (12)
O1—Cu1—N2—C11	−117.8 (2)	C9—C10—C11—N2	−1.1 (3)
N3—Cu1—N2—C11	−8.41 (14)	C11—N2—C12—C8	0.6 (2)
N1—Cu1—N2—C11	178.06 (14)	Cu1—N2—C12—C8	177.78 (11)
O1W—Cu1—N2—C11	82.79 (13)	C11—N2—C12—C1	−178.71 (13)
O1—Cu1—N2—C12	65.5 (2)	Cu1—N2—C12—C1	−1.56 (15)
N3—Cu1—N2—C12	174.87 (9)	C9—C8—C12—N2	−1.4 (2)
N1—Cu1—N2—C12	1.34 (9)	C7—C8—C12—N2	179.62 (14)
O1W—Cu1—N2—C12	−93.92 (10)	C9—C8—C12—C1	177.88 (14)
O1—Cu1—N3—C14	−25.06 (9)	C7—C8—C12—C1	−1.1 (2)
N2—Cu1—N3—C14	166.76 (9)	N1—C1—C12—N2	0.85 (19)
O1W—Cu1—N3—C14	69.72 (9)	C5—C1—C12—N2	179.55 (13)
C2—N1—C1—C5	−1.0 (2)	N1—C1—C12—C8	−178.52 (13)
Cu1—N1—C1—C5	−178.33 (11)	C5—C1—C12—C8	0.2 (2)
C2—N1—C1—C12	177.68 (13)	Cu1—O1—C13—O2	174.42 (13)
Cu1—N1—C1—C12	0.33 (16)	Cu1—O1—C13—C14	−2.72 (16)
C1—N1—C2—C3	0.7 (2)	Cu1—N3—C14—C15	155.66 (11)
Cu1—N1—C2—C3	177.57 (12)	Cu1—N3—C14—C13	28.94 (13)
N1—C2—C3—C4	0.2 (3)	O2—C13—C14—N3	164.17 (14)
C2—C3—C4—C5	−0.8 (3)	O1—C13—C14—N3	−18.54 (18)
N1—C1—C5—C4	0.4 (2)	O2—C13—C14—C15	36.5 (2)
C12—C1—C5—C4	−178.26 (14)	O1—C13—C14—C15	−146.24 (14)
N1—C1—C5—C6	179.43 (14)	N3—C14—C15—O3	−55.86 (17)
C12—C1—C5—C6	0.8 (2)	C13—C14—C15—O3	69.36 (16)
C3—C4—C5—C1	0.6 (2)	N3—C14—C15—C16	63.86 (19)
C3—C4—C5—C6	−178.46 (16)	C13—C14—C15—C16	−170.93 (14)

### Hydrogen-bond geometry (Å, °)

**Table:**

*D*—H···*A*	*D*—H	H···*A*	*D*···*A*	*D*—H···*A*
O1W—H1W1···Cl1	0.80	2.36	3.1411 (13)	166
O1W—H2W1···O2^i^	0.82	1.90	2.7114 (18)	167
O3—H1O3···O1^ii^	0.90	2.03	2.9089 (18)	164
N3—H1N3···Cl1^iii^	0.86	2.62	3.3992 (13)	151
N3—H2N3···O2W^i^	0.94	2.07	3.0085 (19)	175
O2W—H1W2···Cl1^i^	0.87	2.35	3.2104 (16)	175
O2W—H2W2···Cl1^iv^	0.84	2.33	3.1463 (14)	162
O3W—H1W3···O2W	1.01	1.95	2.954 (2)	170
O3W—H2W3···O3^ii^	0.91	2.03	2.901 (2)	159
C7—H7A···O2^v^	0.93	2.41	3.292 (2)	157

Symmetry codes: (i) −*x*+1, −*y*+1, −*z*+1; (ii) −*x*+2, −*y*+1, −*z*+1; (iii) *x*+1, *y*, *z*; (iv) *x*, *y*−1, *z*+1; (v) *x*, *y*, *z*−1.
